# A cross-sectional approach including dog owner characteristics as predictors of visceral leishmaniasis infection in dogs

**DOI:** 10.1590/0074-02760190349

**Published:** 2020-04-27

**Authors:** Ana Izabel Passarella Teixeira, Debora Marcolino Silva, Lúcia Rolim Santana de Freitas, Gustavo Adolfo Sierra Romero

**Affiliations:** 1 Universidade de Brasília, Faculdade de Medicina, Núcleo de Medicina Tropical, Brasília, DF, Brasil; 2 Ministério da Saúde, Secretaria de Vigilância Epidemiológica, Brasília, DF, Brasil

**Keywords:** canine visceral leishmaniasis, risk factors, prevalence, cross-sectional study, serology

## Abstract

**BACKGROUND:**

Visceral leishmaniasis (VL) is relevant for human and animal public health. Several factors have been associated with the risk of *Leishmania infantum* infection in dogs. However, dog owner characteristics have been rarely explored.

**OBJECTIVES:**

To estimate the prevalence and to identify the associated factors for VL infection including dog owners characteristics.

**METHODS:**

A cross-sectional study was conducted including dogs from an endemic canine visceral leishmaniasis (CVL) region in the Federal District, Brazil. The infection was detected using parasitological, serological, and molecular methods. The associated factors were identified through Poisson regression modelling.

**FINDINGS:**

The prevalence of infection was 26.25% [95% confidence interval (CI): 20.05 to 33.57]. The associated factors were: short coat prevalence ratio (PR) = 2.33 (95% CI: 1.02 to 5.22); presence of backyard with predominance of soil and/or vegetation PR = 4.15 (95% CI: 1.35 to 12.77); and highest gross family income score PR = 2.03 (95% CI: 1.16 to 3.54).

**MAIN CONCLUSION:**

This is the first study that relates higher socioeconomic status of dog owners as an independent factor associated with higher prevalence of VL infection, along with other strongly associated factors related to receptive environment for phlebotomines. Our findings strengthen the need for exploration of the biological and behavioural bases linking dog owner characteristics to the risk of canine infection in prospective cohort studies.

American visceral leishmaniasis (VL) is a zoonotic disease associated with infection by *Leishmania infantum*, a parasite transmitted by the bite of phlebotomine females, especially *Lutzomyia longipalpis*.^1^ Human visceral leishmaniasis (HVL) is a prominent public health problem worldwide.^2^ In Brazil, the disease burden has been estimated as 20.9 (UI: 95%, 11.3 to 34.8) age-standardised disability-adjusted life years (DALYs) rate per 100.000 inhabitants.^3^ In 2014, the total cost of VL was estimated as 15 million US dollars, including direct medical costs related to diagnostic, treatment and care provided to patients and indirect costs through productivity loss due to premature mortality and morbidity.^4^

Dogs are considered the main source of infection in urban areas and this represents a major challenge for the implementation of control measures, resulting in the euthanasia of seropositive animals in an attempt to reduce the risk of transmission to humans.^5^ However, euthanasia has been increasingly rejected due to the appreciation of pets, especially dogs.^6^ ^,^ ^7^ In addition, its ineffectiveness has raised concerns.^8^

Because of these difficulties, new preventive and therapeutic measures have been developed and applied in recent years, such as the use of anti-ectoparasite products, repellents and even the treatment of the disease in animals. Although these measures, directly or indirectly, reduce the risk of acquiring the infection or improve the prognosis for the development of symptomatic canine visceral leishmaniasis (CVL), further studies are needed to identify their possible impacts on human health.^9^ ^,^ ^10^ ^,^ ^11^ For these reasons, it is important to study the factors that may be involved in the disease process, improving its understanding, which may contribute to better strategies for disease control.

Indeed, several studies investigated the factors, especially biological characteristics of dogs, associated with the risk of developing CVL. A short coat, for example, stands out as the most relevant factor.^12^ ^,^ ^13^ ^,^ ^14^ However, there are multiple other potential factors that have not been explored yet.

These include (i) the owner’s knowledge and awareness of VL, both in humans and dogs,^15^ ^,^ ^16^ (ii) the availability of adequate sanitary systems, such as sewage, treated water and garbage collection,^13^ ^,^ ^17^ (iii) the presence of organic matter, either in the yard or in some proximity,^18^ and (iv) the existence of highways and the movement of people and animals.^19^ It should be emphasised that all of these studies demonstrate that human action on the environment is closely related to the development of the disease.

To ascertain human action in the occurrence of CVL, we evaluated the prevalence of CVL in an endemic region in the Federal District and explored the association of several risk factors for infection, including the less studied risk factors which are related to the socioeconomic status of the dog owners and the care provided to the animals.

## MATERIALS AND METHODS

*Type of study* - Cross-sectional descriptive and analytical study.

*Period of study* - The study was conducted from October 2015 to March 2017.

**Fig. 1:**
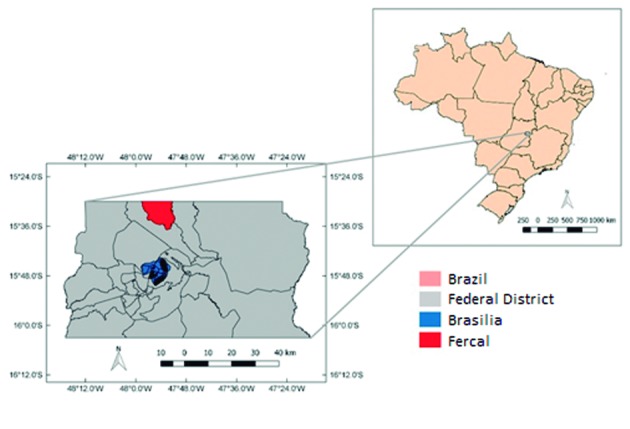
geographical location of the Fercal Administrative Region in Federal District, Brazil.

*Location of study* - Data collection was performed in the XXXI administrative region of the Federal District, named Fercal. This location is endemic for human and canine cases of VL^20^ ^,^ ^21^ (Fig. 1).

*Participants of the study* - The dog owners participated in the study by completing a questionnaire for the assessment of their socio-economic status and the care they provide to their dogs. The dogs included in the study were physically examined. In addition, blood, bone marrow, or both were collected from the animals for diagnostic tests

*Eligibility criteria* - The eligibility criteria were applied by residence and the unit of analysis was the dog. Thus, the residence was eligible when owner’s and dogs’ eligibility criteria were met. The owner’s criteria were being older than 18 years old and willingness to read and sign the Informed Consent Form. The dogs’ criterion was being older than four months. Animals with behaviour or health problems that precluded the application of diagnostic procedures under mild sedation, such as severe heart disease, seizures and extreme aggression, were excluded.

*Sample size* - The canine population in the Fercal administrative region was estimated based on the human population of 8746 inhabitants, which was estimated from a sample survey conducted in 2013.^22^ It was assumed that every household was composed of five inhabitants. Because no official data of dog population was available at that time, we performed preliminary non-systematic observations in the study field and presumed that 20% of the households would have a dog, resulting in the estimated population of 342 dogs.

Based on previous studies in which the seroprevalence for CVL in Fercal ranged from 7 to 14%,^9^ ^,^ ^22^ the expected frequency of canine infection was estimated as 10%. Considering a margin of error of 5% and a confidence interval (CI) of 95%, and accounting for a possible conglomerate effect (a residence may have more than one dog), a correction in the design effect of 1.5 was included in the calculation.^23^ Losses of 10% were also estimated resulting in a final sample size of 160 animals. Samples were calculated using the *Stat Calc* program from Epi Info, version 7.2.0.1.

*Sampling* - The data collection was performed in a systematic manner starting with the residence in Fercal closest to the *Plano Piloto* of the Federal District; data were collected from only one house from each block. In cases where the residence approached first was not eligible, the team would visit the next house, and so on, until an eligible residence was found. After the data collection was completed at an eligible residence, the team continued the process in the next block. There were blocks in which no animals were found and blocks in which none of the owners agreed to participate in the study. Accordingly, no data was collected from such blocks. All blocks were covered by the collection team so that the entire Fercal territory was represented in the sampling [Supplementary data (Fig. 1)].

**Fig. 2:**
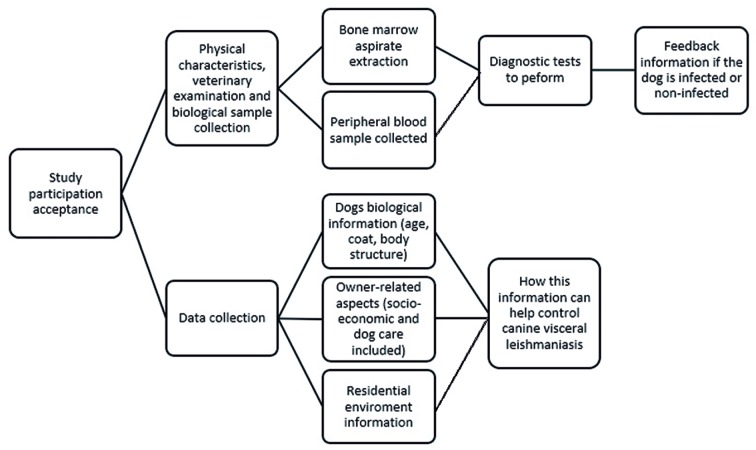
study stages flowchart as presented to the owners.

*Data collection* - After verifying compliance with the eligibility criteria, the team presented the study to the owners and provided details on the process and data collection (Fig. 2). After being informed that positive results for leishmaniasis would be reported to the Diretoria de Vigilância Ambiental do Distrito Federal (Environmental Surveillance Board of the Federal District; DIVAL-DF) for the implementation of the adequate control measures, the owners read and signed the informed consent form.

The owners filled out a questionnaire about several aspects of their residence and the care provided to the dogs. Also, a series of questions were asked to estimate their socioeconomic status, according to recommendations by the Associação Brasileira de Empresas de Pesquisa (Brazilian Association of Research Companies; ABEP)^24^ (Table I).

**TABLE I:** Relationship between economic class and mean gross family income^(24)^

Class	Points	Mean gross family income in Brazilian Reais (BRL)
A1	42-46	11,037
A2	35-41	6,006
B1	29-34	3,118
B2	23-28	1,865
C1	18-22	1,277
C2	14-17	895
D	8-13	895
E	0-7	895

The data including the physical characteristics and clinical data of the dogs were recorded. The dogs were categorised as asymptomatic or symptomatic according to the *LeishVet Guidelines*.^25^ Clinical data were recorded without knowledge of the infection diagnosis, and a score of signs was assigned to each dog following a model adapted from Proverbio et al.^26^ This adaptation consisted of eliminating items that could not be determined by a single physical examination. The excluded items were change in appetite, change in mental state, exercise intolerance, weight loss, polyuria, polydipsia and proteinuria. These items, if present at their highest intensity, could add up to 20 points to the score.

Because there was more than one veterinarian performing biological sample collection and physical examination, other items that could be subjectively evaluated were modified so that only the presence or absence of these signs was reported. These items were skin lesions, hepatosplenomegaly, epistaxis, vomiting, diarrhea, claudication, altered pigmentation, hyperkeratosis and onychogryphosis. With this modification, these items could add 21 points if each factor was rated as maximum intensity. The modified clinical score was validated in the field through a pilot study in which 20 evaluations were performed by two veterinarians. The final evaluation applied in the study had a maximum score of 46 points, instead of 87 as in the original evaluation according to Teixeira et al.^27^

*Collection, transport, and storage of biological samples* - Peripheral blood (cephalic vein, saphenous vein or jugular vein) was collected using a 3 mL syringe and a 25 × 0.7 mm needle. The collected blood was stored in two tubes, one with EDTA anticoagulant for molecular tests and the other without anticoagulant to perform serological tests.

The samples were transported to the Leishmaniasis Laboratory of the Tropical Medicine Centre/University of Brasilia (NMT/UnB) under refrigeration, i.e. at 4-8ºC. Immediately after arrival at the laboratory, serum was separated by centrifugation at 1512 × *g* for 3 min. Whole blood and serum samples were stored at -20ºC until the serological or molecular tests were performed.

The collection of bone marrow aspirate from the sternum was performed under sedation. Sedation was performed with intramuscular injection of ketamine (8 mg per kg body weight) combined with acepromazine (0.1 mg per kg body weight). After sedation, the animals were placed on a stainless-steel table for trichotomy and sequentially cleaned with soap and water, iodinated alcohol and 70% alcohol. The collection was performed with a disposable syringe (20 mL) and a 40-gauge needle (1.2 mm).

*Definition of infection* - A dog with positive results for any of the following diagnostic protocols was considered infected: direct parasitological examination; culture of the parasite; polymerase chain reaction (PCR) amplification of the ITS1 target; Dual-Path Platform rapid test (TR DPP) serology (Biomanguinhos®) and ELISA serology (EIE-CVL) (Biomanguinhos®), sequentially; and (RT DPP) (Biomanguinhos®) serology and ELISA serology (rK39), sequentially. Dogs that presented negative results for all diagnostic protocols mentioned above were considered non-cases.

*Diagnostic tests procedures* - Direct parasitology; parasite culture; PCR amplification of the ITS1 target; RT DPP serology (Biomanguinhos®) and ELISA serology (EIE-CVL) (Biomanguinhos®), sequentially; TR DPP serology (Biomanguinhos®) and ELISA serology (Rk39), sequentially, were performed according to the methodology described in the study by Teixeira et al.^27^ When the tests were performed, the professionals were not aware of the identity of the source animals or of the results obtained for the other tests.

*Data management* - All missing data were appointed in the frequency and proportions analyses. Data were collected as reported by the owner. However, the data was verified where possible, for example through vaccination cards or visual examination. All data collected, including biological samples, were coded so that the analyses were conducted blindly.

*Statistical analysis* - Descriptive analyses of the data were performed, with calculations of disease/infection prevalence and 95% CI. Subsequently, the analytical phase was initiated with a bivariate analysis and respective prevalence ratio (PR), in which the significant variables with p-values less than or equal to 0.2 were selected for the multivariate analysis using Poisson regression.

For these analyses, the calculation of the quartile analysis of the clinical signs scores suggestive of CVL and of the family income score and its transformation into a categorical variable can be seen in Table II.

Poisson regression with robust variance was used to estimate PR and respective 95% CI as measures of association. Modelling followed a hierarchical structure in which owner characteristics and care of the dogs were considered as proximal exposures, characteristics of the residential environment as an intermediate exposure, and biological factors as distal exposures.

**TABLE II:** Absolute and relative frequencies of clinical signs observed in infected and uninfected dogs residing in the Fercal administrative region, Federal District, Brazil, 2015-2017

Findings		Infected	Non-infected	p-value
Bodily condition	Obese/Normal	35/42 (83.33%)	99/116 (85.34%)	0.131
	Thin	3/42 (7.14%)	14/116 (12.06%)	
	Cachectic	4/42 (9.53%)	3/116 (2.58%)	
Mucosal paleness	None	34/42 (80.95%)	98/116 (84.48%)	0.597
	Present	8/42 (19.05%)	18/116 (15.52%)	
Dehydration	None	40/42 (95.24%)	113/116 (97.41%)	0.926
	Mild	1/42 (2.38%)	2/116 (1.71%)	
	Moderate to intense	1/42 (2.38%)	1/116 (0.85%)	
Mild muscle atrophy of the limbs	None	41/42 (97.62%)	115/116 (99.14%)	0.451
	Present	1/42 (2.38%)	1/116 (0.85%)	
Skin lesions	None	29/42 (69.05%)	80/118 (68.96%)	0.992
	Present	13/42 (30.96%)	36/116 (31.04%)	
Hepatosplenomegaly	None	35/40 (87.50%)	107/116 (92.24%)	0.366
	Present	5/40 (12.50%)	9/116 (7.76%)	
Conjunctivitis and/or Keratitis	None	35/42 (83.33%)	107/116 (92.24%)	0.247
	Unilateral and mild	2/42 (4.76%)	2/116 (1.72%)	
	Severe unilateral/bilateral	5/42 (11.90%)	7/116 (6.03%)	
Uveitis and/or Blepharitis	None	39/42	110/115	0.481
	Present	3/42 (7.14%)	5/115 (4.35%)	
Lymph adenomegaly	None	24/42 (57.14%)	73/116 (62.93%)	0.803
	1 to 2 lymph nodes	12/42 (28.57%)	29/116 (25.0%)	
	3 or more / widespread	6/42 (14.28%)	14/116 (12.07%)	
Mouth ulcers or nodules	None	41/42 (97.62%)	116/116 (100.0%)	0.096
	Present	1/42 (2.38%)	0/116 (0%)	
Diarrhea	None	39/42 (92.86%)	111/116 (95.69%)	0.473
	Present	3/42 (7.14%)	5/116 (4.31%)	
Claudication	None	42/42 (100.0%)	115/116 (99.14%)	0.546
	Present	0 (0%)	1/116 (0.85%)	
Erythema (1 to 25% of the body surface)	None	42/42 (100.00%)	114/116 (98.28%)	0.392
	Present	0 (0%)	2/116 (1.72%)	
Dry exfoliative dermatitis	None	32/42 (76.19%)	94/116 (81.03%)	0.739
	1 to 25% of the body	7/42 (16.67%)	13/116 (11.21%)	
	> 25 to 40% of the body	2/42 (4.76%)	4/116 (3.44%)	
	> 40% of the body	1/42 (2.38%)	5/116 (4.31%)	
Ulcerative dermatitis	None	38/42 (90.48%)	112/116 (96.55%)	0.306
	1 to 25% of the body	3/42 (7.14%)	3/116 (2.58%)	
	> 25% of the body	1/42 (2.38%)	1/116 (0.85%)	
	-	-	-	
Nodular dermatitis	None	39/42 (92.86%)	114/116 (99.24%)	0.086
	Present	3/42 (7.14%)	2/116 (1.72%)	
Pustular dermatitis	None	41/42 (97.62%)	115/116 (99.24%)	0.451
	Present	1/42 (2.38%)	1/116 (0,86%)	
Alopecia	None	36/42 (85.71%)	105/116 (90.52%)	0.766
	1 to 25% of the body	3/42 (7.14%)	8/116 (6.89%)	
	> 25% of the body	3/42 (7.14%)	3/116 (2.59%)	
Altered pigmentation	None	40/42 (95.24%)	111/116 (95.69%)	0.903
	Present	2/42 (4.76%)	5/116 (4.31%)	
Hyperkeratosis of truffles and cushions	None	39/42 (92.86%)	114/116 (98.28%)	0.086
	Present	3/42 (7.14%)	2/116 (1.72%)	
Onychogryphosis	None	38/42 (90.48%)	114/116 (98.28%)	0,023
	Present	4/42 (9.52%)	2/116 (1.72%)	

*Ethical considerations* - All procedures were designed to reduce animal suffering. All owners were informed about the risks of the procedures and the risks of VL, both for the human and canine populations, and the owners had access to the results of the tests. The study was approved by the Ethics Committee on Animal Use of UnB under the number UnBDoc 11253/2015, in accordance with Law 11.794 of October 8, 2008.^28^

## RESULTS

A total of 160 dogs belonging to 112 owners were included in the study (1.47 dogs per owner on average). Among the 112 owners, 76 (67.9%) had one participating dog, 26 (23.2%) had two participating dogs, and 10 (8.9%) had more than two participating dogs. There were 28 refusals; additionally, five dogs were excluded for having severe heart disease, and one dog was excluded for having been vaccinated against VL. Of the questionnaires used to obtain data regarding owners, residences and care offered to dogs, 109 were considered valid, and three invalid.

The mean gross family income score was 17.45 points (SD = 5.70). Most owners belonged to socioeconomic strata C1 and C2, with a mean gross family income of BRL 895.00 to BRL 1,277.00. The PR in each quartile was: 0.58 (95% CI: 0.25 to 1.30) for quartile 1, 0.73 (95% CI: 0.34 to 1.55) for quartile 2, 1.47 (95% CI: 0.95 to 2.27) for quartile 3, and 1.08 (95% CI: 0.57 to 2.06) quartile 4. Of the 160 dogs, 71 (44.4%) were females, and 89 (55.6%) were males. The mean age was 3.06 years (SD 2.86 years, range four months to 15 years).

Clinical signs data were available for 158 dogs, of which 53.79% (85/158) were asymptomatic (score less than or equal to 1 point) and 46.21% (73/158) were symptomatic (score greater than or equal to 2). The clinical score values for the infected dogs with onychogryphosis were 6, 11, 14 and 26. The clinical score values for the dogs without infection were 3 and 4. The frequency of clinical signs observed in infected and noninfected dogs is described in Table II.

Bone marrow aspirate was collected from 62.5% (100/160) of the dogs. Of those samples, 2% (2/100) were positive for isolation of the parasite in culture and 8% (8/100) were positive by direct observation of the parasite.

Eighty-eight (55%) of the 160 blood samples and 56 (56%) out of the 100 bone marrow samples were positive for kDNA PCR. These positive samples were subjected to PCR for amplification of ITS1; 14/88 (15.90%) of the whole blood samples and 13/56 (23.21%) of the bone marrow samples were positive. Thus, 26/160 animals (16.25%) were considered positive according to the parameters used to define infection using molecular diagnostic tests. Of these, 12 animals were positive only using bone marrow analysis, 13 animals were positive only using peripheral blood analysis, and one animal was positive using both of these.

Regarding the serological tests, 25/160 animals were positive by RT DPP (Biomanguinhos®) and 24/160 by EIE-CVL (Biomanguinhos®). However, only 15 (9.38%) animals were positive by both tests. In turn, when RT DPP (Biomanguinhos®) and ELISA rk39 were used in combination, only 19 (11.88%) animals were positive by both tests. Therefore, considering the criteria established in the methodology section for the definition of infection using serological methods, 42 infected dogs were identified.

**Fig. 3:**
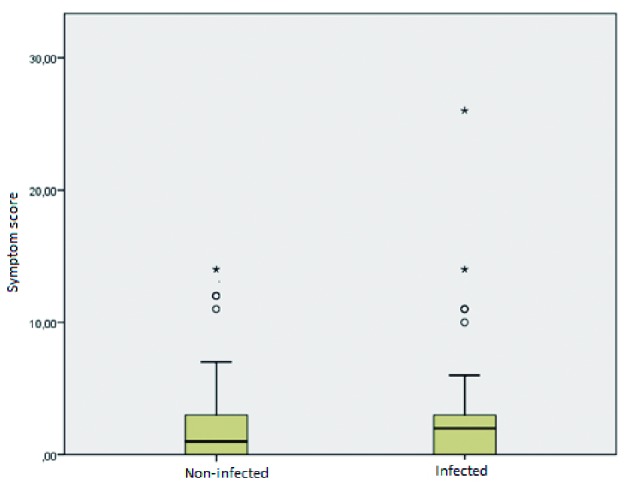
distribution of the clinical canine visceral leishmaniasis score in the infected and uninfected animal groups, Brasília 2017. 118 uninfected dogs, median 1 (IQR: 0-3, range 0-14 points). 42 infected dogs, median 2; (IQR: 0-3, range 0-26 points). (Mann-Whitney U test; p = 0.377).

The estimated prevalence of infected dogs was 26.25% (95% CI: 20.05 to 33.57). Of the 42 infected dogs, only 22 presented a score of signs compatible with CVL greater than or equal to 2 and were considered symptomatic. Therefore, the prevalence of disease in the studied sample was 13.75% (95% CI: 9.26 to 19.94). There was no statistically significant difference in the median of the clinical score among infected and noninfected dogs (Fig. 3).

The estimated seroprevalence using sequential TR DPP/EIE-CVL testing was 15/160 (9.38%) animals (95% CI: 5.76 to 14.89). The estimated seroprevalence using sequential RT DPP/ELISA rK39 testing was 19/160 dogs or 11.88% (95% CI: 7.74 to 17.80).

Table III shows the bivariate analysis of the biological factors possibly associated with CVL. Only two factors (short coat and light-colour coat) exhibited at least a moderate statistical association (p-value ≤ 0.2). Table IV provides the results of the bivariate analysis of the factors that were related to the environment in which the animal resided and were associated with CVL. All factors exhibited a moderate statistical association (p-value ≤ 0.2).

**TABLE III:** Bivariate analysis of the biological factors associated with canine visceral leishmaniasis in dogs living in residence in the administrative region of Fercal, Federal District, Brazil, 2015-2017

Variables	Categories	N	Exposure	Proportion of infected dogs among the exposed dogs	Proportion of infected dogs among nonexposed dogs	p value^*^	Prevalence ratio (95% CI)
Sex	0-Male 1 - Female	160	Being female	19/71 (26.7%)	23/89 (25.8%)	0.896	1.04 (0.61 to 1.74)
Coat	0- Non-short coat 1- Short coat	155	Having short coat	37/119 (31.1%)	5/36 (13.9%)	0.042	2.24 (0.90 to 5.27)
Coat colour	0 - Non-light colour 1 - Light-colour	143	Having light-colour coat	18/50 (36.0%)	20/93 (21.5%)	0.061	1.67 (0.98 to 2.86)
Body structure	0 - Normal or obese 1 - Any degree of thinness	160	Being thin	7/20 (35.0%)	35/140 (25.0%)	0.342	1.40 (0.72 to 2.72)
Animals with clinical signs > 2 points	0 - Up to 1 point 1 - Greater than 2 points	158	Having 2 or more clinical score points	22/73 (30.1%)	19/85 (22.4%)	0.266	1.35 (0.80 to 2.29)

*: chi-square test.

**TABLE IV:** Bivariate analysis of the factors related to the household environment in which the animal resides and associated with visceral leishmaniasis in the administrative region of Fercal, Federal District, Brazil, 2015-2017

Variables	Categories	N	Exposure	Proportion of infected dogs among the exposed dogs	Proportion of infected dogs among nonexposed dogs	p value^*^	Prevalence ratio (95% CI)
Presence of other animals (any species)	0 - no 1 - yes	156	Dog whose owner declared that his pet lives with other animals	28/116 (24.1%)	14/40 (35.0%)	0.182	0.69 (0.41 to 1.17)
Having a backyard in a residential area	0 - no 1 - yes	156	The dog lives in a residence with a backyard.	41/143 (28.7%)	1/13 (7.7%)	0.188	3.73 (0.56 to 24.94)
Backyard area with a predominance of land and / or vegetation	0 - no 1 - yes	156	The dog lives in a residence with a backyard with a grassy area or land	39/117 (33.3%)	3/39 (7.7%)	0.002	4.33 (1.42 to 13.24)
Presence of accumulated organic matter in the backyard	0 - no 1 - yes	156	Presence of organic matter in the backyard (plant debris, organic waste)	23/70 (32.9%)	19/86 (22.1%)	0.132	1.49 (0.88 to 2.50)
Residence with screened windows	0 - no 1 - yes	156	The residence has screened windows	3/21 (14.3%)	39/135 (28.9%)	0.160	0.49 (0.17 to 1.48)

*: chi-square test.

The results of the bivariate analysis of the exposure factors that were related to animal care performed by owners and associated with CVL revealed that all factors had a statistically significant association (p-value ≤ 0.2). Notably, the vaccination cards for the dogs were confirmed (Table V).

Poisson regression model was used with the exposure factors that exhibited at least a moderate statistical association in the bivariate analysis (p ≤ 0.2). In the model, according to Table VI, the factors associated with higher prevalence of infection were short coat, backyard area with a predominance of land and/or vegetation and highest family gross income score.

**TABLE V:** Bivariate analysis of factors related to animal care by their owners and associated with canine visceral leishmaniasis in the administrative region of Fercal, Federal District, Brazil, 2015-2017

Variables	Categories	N	Exposure	Proportion of infected dogs among the exposed dogs	Proportion of infected dogs among non-exposed dogs	p value^*^	Prevalence ratio (95% CI)
Animal was vaccinated against any disease.	0 - no 1 - yes	156	Having received any dose of vaccine to prevent any disease	30/127 (23.6%)	12/29 (41.4%)	0.052	0.57 (0.33 to 0.97)
Owner knows symptoms of VL in humans	0 - negative or ignored 1 - yes	155	Animal whose owner declared to knowing what are the HVL symptoms	8/17 (47.1%)	34/138 (24.6%)	0.127	1.91 (1.07 to 3.42)
The owner showed up with the dog in at least one consultation with the veterinarian in the past year	0 - no 1 - yes	155	Animal whose owner stated that he or she took the dog to at least one consultation with a veterinarian in the past year	4/31 (12.9%)	37/124 (29.8%)	0.056	0.43 (0.17 to 1.12)
Highest gross family income score	0 - up to 16 points 1 - >16 points	156	Having an earning power score of 16 points or more	29/89 (32.6%)	13/67 (19.4%)	0.066	1.68 (0.95 to 2.98)

*: chi-square test.

**TABLE VI:** Poisson regression analysis with care factors as a proximal exposure category and biological factors as a distal exposure category

Variables	Non-adjusted prevalence ratio (95% CI)	Adjusted prevalence ratio (95% CI)	p value
Coat (short)	2.24 (0.91-5.27)	2.33 (1.02-5.22)	0.044
Backyard area with a predominance of land and/or vegetation	4.33 (1.42-13.24)	4.15 (1.35-12.77)	0.013
Highest family gross income score	1.68 (0.95-2.98)	2.03 (1.16-3.55)	0.014

## DISCUSSION

The prevalence of infection identified in the present study was higher (26.25%; 95% CI: 20.05 to 33.57) than that previously recorded in the endemic areas of the Federal District and higher than that estimated a priori during the sample calculation. In previous studies, the reported prevalence ranged from 7 to 14%.^22^ ^,^ ^29^ This discrepancy is most likely due to the combined use of a larger number of diagnostic techniques in the present study, especially PCR, which enabled the detection of a larger number of infected animals because of its greater sensitivity.^27^ ^,^ ^30^

The difference of 12.5% between the prevalence of infection (26.25%; 95% CI: 20.05 to 33.57) and the prevalence of the disease (13.75%; 95% CI: 9.26 to 19.94) may have resulted from the greater sensitivity of the combination of diagnostic methods used for the detection of infected dogs. The detection of asymptomatic-infected dogs is important in terms of epidemiological surveillance, especially because autochthonous cases of human VL have been reported in this region.^20^ ^,^ ^21^ Issues related to the detection of infected animals are even more concerning when assessing the seroprevalence of infection based on the combination of results from the TR DPP/EIE-CVL tests,^31^ currently recommended by a disease control program (9.38%; 95% CI: 5.76 to 14.89), which were markedly lower than the estimate obtained by combined methodologies that included molecular diagnosis. The under-detection of cases of asymptomatic canine infection caused by the lack of sensitivity of the methods recommended for routine examination has already been highlighted by other authors as a possible factor associated with the lack of effective CVL control measures currently recommended in Brazil.^27^ ^,^ ^32^ ^,^ ^33^ ^,^ ^34^

There was no significant difference between the clinical signs scores for infected and noninfected dogs, and there were dogs with suggestive symptoms that were not infected with leishmaniasis but were possibly affected by other diseases that were not specifically investigated in this study. This observation is consistent with the perception that the clinical syndrome associated with CVL is nonspecific. Onychogryphosis was the only clinical finding that was significantly associated with CVL. This finding is consistent with the literature, which reports that onychogryphosis is one of the clinical signs most frequently associated with dogs with VL.^25^ ^,^ ^35^

Poisson regression with robust adjustment was used to explore the relationship between exposure factors and infection outcome, and proximal and distal exposure categories were established for the development of the model. The use of this technique has been indicated in cross-sectional studies with PR because it produces an adequate fit without being affected by the magnitude of the prevalence of the event of interest.^36^

A short coat is a characteristic that has been associated with the risk of CVL in other studies, probably because short-coated dogs attract more vectors than do long-coated dogs probably because of the pattern of body heat radiation or CO_2_ release.^12^ The study by Coura-Vital et al.,^13^ for example, identified a hazard ratio (HR) of 1.9 (95% CI: 1.1 to 3.4), and Leal et al.^14^ estimated an OR of 1.8 (95% CI: 1.5 to 2.1) for the association between short coat and the presence of *Leishmania* infection.

The association of infection with the presence of a backyard at the residence with a predominance of land and/or vegetation reinforces the idea that environmental characteristics are important in the transmission and maintenance of this disease consistently identified in other studies.^37^ ^,^ ^38^ Interestingly, environmental interventions directed to the modification of the scenario around the residences may be impractical because they could have a negative externality, such as reduced plant cover, or reduced soil drainage which creates challenges when determining interventions aimed at protecting dogs from vector bites. Thus, the use of collars with repellents has been recently investigated, with promising results.^9^ ^,^ ^11^ ^,^ ^39^ ^,^ ^40^ ^,^ ^41^ In turn, other interventions that improve the environment, such as the adequate collection of solid waste,^13^ ^,^ ^17^ ^,^ ^42^ which has a positive externality, should always be encouraged among the recommended measures to reduce the infection burden in the community; however, there is a lack of specific studies that determine the magnitude of its potential impact on the transmission of CVL.

Although HVL has been traditionally associated with poverty,^2^ in the present study, there was a significant association between higher family gross income of owners and the possibility of having CVL. This is an association that had not been previously described in the literature. The family gross income score distribution in sample of owners included in the study demonstrated that most of them belonging to socioeconomic class C1 or lower [Supplementary data (Fig. 2)] and the exploration of risk by quartiles show a cut-off for increased risk around the score level between B2 and C1 classes. Then, the external validity of our results related to tutors’ income level would be limited to populations with similar income distribution. The hypotheses that would explain this association may be related to the fact that owners with better socioeconomic status provide better care to animals, which would result in a higher probability of survival in relation to other lethal and vaccine-preventable conditions (*e.g.*, distemper virus and parvovirus);^43^ as seen, the symptomatic dogs were from owners with a lower income and the asymptomatic dogs were from a bigger income. Additionally, when the animal ages, these diseases could trigger some immunodeficiency. Therefore, increased survival time increases the exposure risk of VL infection as well as the susceptibility of the animal. Furthermore, better nutrition could keep the dog healthier, and even if infected by leishmaniasis, the dog would survive longer. This association can be very challenging in the current framework of recommendations, which include the euthanasia of infected dogs, as owners with better income may be those that most often refuse euthanasia.^44^ This lack of euthanasia allows dogs to remain in the environment as sources of the disease to other dogs and to the human population. The present observation related to the socioeconomic status of the owners contributes to raising new hypotheses to explain the lack of effectiveness of current control models that have failed to prevent the territorial dispersion of the disease.

In epidemiological terms, the role of asymptomatic dogs as a source of infection may be even more important than the role of the population of sick dogs, which are at a lower proportion, a fact that has been addressed in other studies.^45^ ^,^ ^46^ Although asymptomatic dogs transmit *Leishmania* spp*.* at a lower rate than do symptomatic dogs,^32^ ^,^ ^47^ they may represent a numerically larger group of animals serving as source of infection. Moreover, symptomatic dogs that traditionally undergo interventions, whether euthanasia or treatment, constitutes a smaller portion of the population, as demonstrated in this study.

The use of the owner’s income score as an exposure factor opens a debate for future studies on the role of owners in the process of CVL prevention; until now, there has been an almost exclusive focus on the actions of epidemiological surveillance teams dedicated to the management of zoonoses. The success of new prophylactic interventions, such as the use of insecticide-impregnated dog collars, oral repellents or even vaccines, will certainly depend on the participation of owners to ensure the appropriate use and timely replacement of collars or the administration of other drugs and vaccines, which can have a direct or indirect relationship with the socioeconomic status of the owners, influencing the effectiveness of the interventions.

This study has limitations inherent to a cross-sectional approach, such as the inability to ascertain causality. The limitation imposed by the absence of laboratory tests complementary to the clinical data should also be considered, which could have helped in the identification of some effects of infection on kidney function, haematological conditions and the nutritional status of apparently healthy animals. The possibility of observation bias should also be considered, as several observers evaluated and recorded the clinical conditions of the studied animals and applied the questionnaires. In this sense, efforts were made to minimise the observation bias in the clinical evaluation, such as the execution of a previous pilot project,^27^ as well as masking of the clinical data to perform diagnostic techniques. The generalisation of these results is something to be viewed with precaution due to the type of study that was conducted. However, the explanatory hypotheses for the results deserve attention and should be the object of further cohort research.

In addition to the aforementioned limitations, the possibility of survival bias should be considered when understanding the significance of the highest income score associated with a higher prevalence of CVL. That is, all the hypotheses discussed here may have been affected by a higher probability of survival when a dog has a guardian with higher income. This does not diminish the importance that asymptomatic dogs could have in the maintenance of VL in a community. Therefore, future studies should be methodologically careful when making this distinction. Finally, we estimated dog population size corresponding to 4% of the human population. Actually, that number is lower than the usual dog to human ratio reported in the literature from other localities in Brazil and other countries. In 2019, the public health authorities in the Federal District estimate dog population equivalent to 12% of the human population for rabies vaccination purposes.^48^ Besides the number of dogs in the studied region, the actual number of dogs recruited in our study resulted in acceptable precision of the prevalence rates and PR estimates.

*In conclusion* - The present study identified three relevant factors associated with *Leishmania* infection in a predominantly asymptomatic canine population, standing out as the first study to associate owners’ higher socioeconomic status with the highest prevalence of infection.
