# Supplementary material for: A cross-sectional approach including dog owner characteristics as predictors of visceral leishmaniasis infection in dogs
Source: Mem Inst Oswaldo Cruz. 2020 Apr 27;115:e190349. doi: 10.1590/0074-02760190349 (PMC7184770; doi:10.1590/0074-02760190349)
Supplement: Supplementary file 1 [file 1678-8060-mioc-115-e190349-s.pdf]

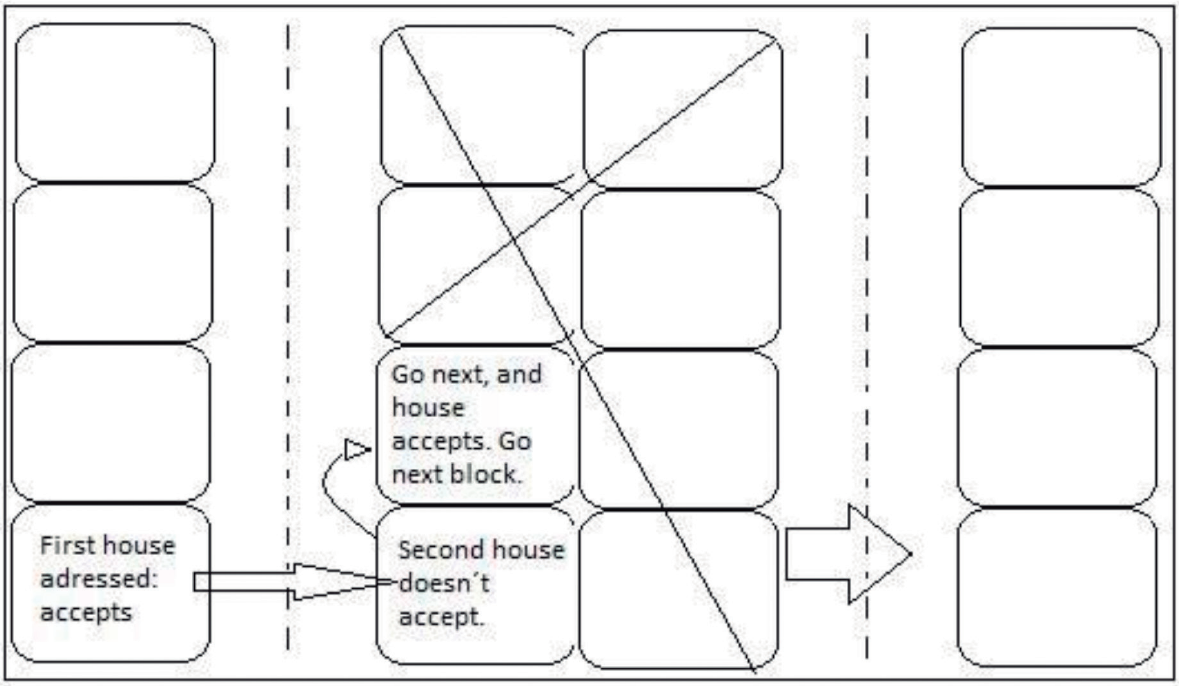

Fig. 1: representation of how the systematic sampling was performed.

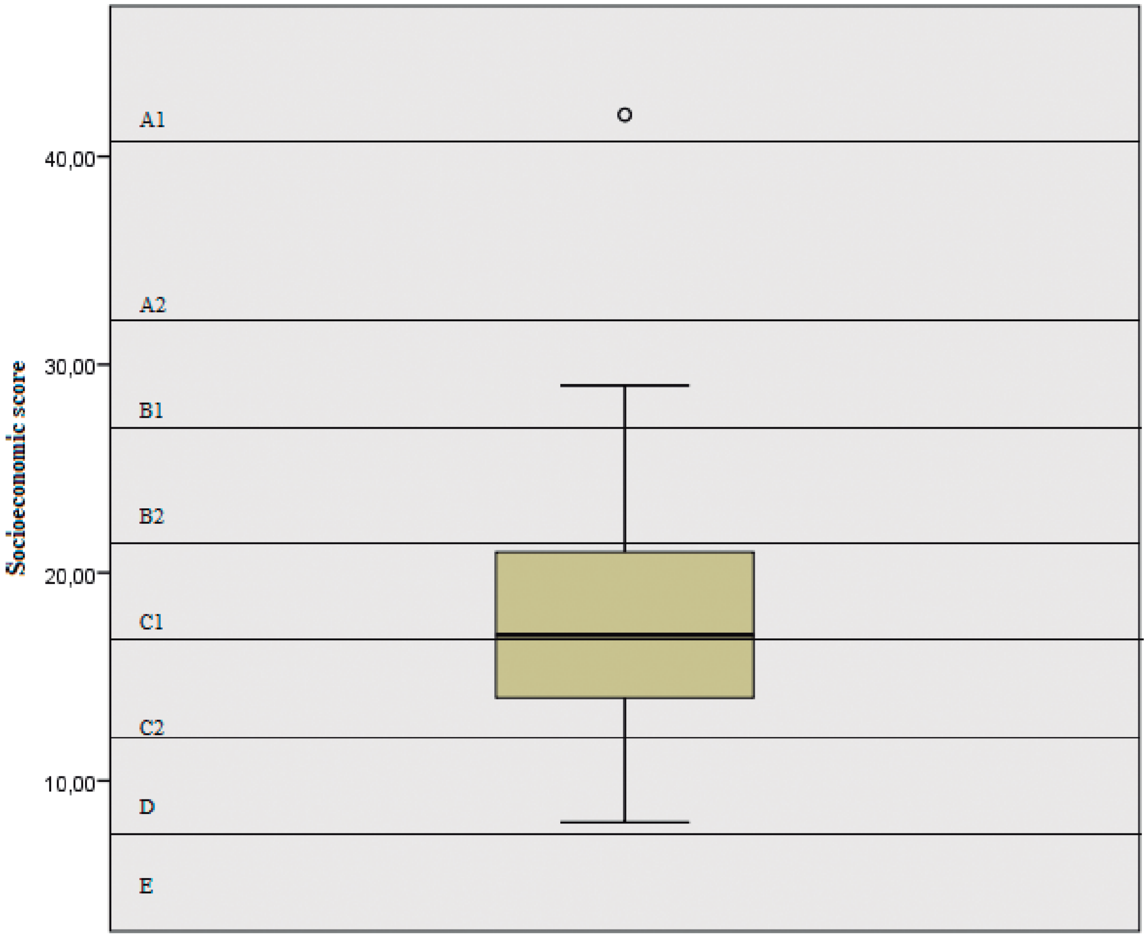

Fig. 2: family gross income score distribution in a sample of 112 dog owners in the Fercal Administrative Region in Federal District, Brazil.
